# The TSC1-TSC2 complex consists of multiple TSC1 and TSC2 subunits

**DOI:** 10.1186/1471-2091-13-18

**Published:** 2012-09-24

**Authors:** Marianne Hoogeveen-Westerveld, Leontine van Unen, Ans van den Ouweland, Dicky Halley, Andre Hoogeveen, Mark Nellist

**Affiliations:** 1 Department of Clinical Genetics, Erasmus Medical Centre, Dr. Molewaterplein 50, Rotterdam, 3015 GE, The Netherlands

**Keywords:** TSC1, TSC2, TSC1-TSC2 complex, Quaternary structure

## Abstract

**Background:**

Mutations to the *TSC1* and *TSC2* genes cause the disease tuberous sclerosis complex. The *TSC1* and *TSC2* gene products form a protein complex that integrates multiple metabolic signals to regulate the activity of the target of rapamycin (TOR) complex 1 (TORC1) and thereby control cell growth. Here we investigate the quaternary structure of the TSC1-TSC2 complex by gel filtration and coimmunoprecipitation.

**Results:**

TSC1 and TSC2 co-eluted in high molecular weight fractions by gel filtration. Coimmunoprecipitation of distinct tagged TSC1 and TSC2 isoforms demonstrated that TSC1-TSC2 complexes contain multiple TSC1 and TSC2 subunits.

**Conclusions:**

TSC1 and TSC2 interact to form large complexes containing multiple TSC1 and TSC2 subunits.

## Background

Mutations in either the *TSC1* gene on chromosome 9q34 [1], or the *TSC2* gene on chromosome 16p13.3 [2] cause tuberous sclerosis complex (TSC) [3] and lymphangioleiomyomatosis (LAM) [4]. TSC is an autosomal dominant disorder characterised by neurological symptoms, such as epilepsy and autism, and the development of hamartomas in a variety of organs and tissues, including the brain, skin and kidneys. LAM is a progressive lung disease in women caused by abnormal smooth muscle cell proliferation. The *TSC1* and *TSC2* gene products, TSC1 and TSC2, interact to form a protein complex [5]. The TSC2 N-terminal region and multiple regions of TSC1 are important for the TSC1-TSC2 interaction [6,7]. TSC2 contains a GTPase activating protein (GAP) domain, and the TSC1-TSC2 complex is the GAP for the ras homolog expressed in brain (RHEB) GTPase [8]. RHEB-GTP activates the target of rapamycin (TOR) complex 1 (TORC1), resulting in phosphorylation of TORC1 targets, including p70 S6 kinase and the elongation factor 4E binding proteins [9]. Although the TSC2 GAP domain is of obvious functional importance, the role of TSC1 is less clear [10]. TSC1 stabilizes TSC2 [11] and may be important for recruiting TSC2 to membranes [12]. It is also possible that TSC1 facilitates the formation of TSC1-TSC2 complexes containing multiple TSC1 and TSC2 subunits. TSC1-TSC2 oligomers would allow more sensitive allosteric control of TSC1-TSC2 activity [13], as well as providing stability and protection against denaturation [14].

Previously, to investigate the structure of the TSC1-TSC2 complex we performed gel filtration on detergent extracts of cultured cells, and estimated the size of the TSC1-TSC2 complex as > 450 kDa [15]. However, we have been unable to reproduce these results. In the present study, gel filtration experiments using cell extracts obtained by hypotonic lysis [12] indicate that TSC1 and TSC2 form complexes up to ~1 MDa in size, suggesting that our previous analysis underestimated the size of the TSC1-TSC2 complex. In line with our new findings and other results [5,16], immunoprecipitation experiments indicated that TSC1-TSC2 complexes contain multiple TSC1 and TSC2 subunits.

## Methods

### Constructs and antisera

An overview of the TSC1 and TSC2 proteins expressed during this study is given in Additional file 1. To express V5-tagged TSC1, the *TSC1* open reading frame (ORF) was cloned as a NheI-KpnI fragment into pcDNA3.1/V5-His (Invitrogen, Paisley, U.K.). The Tobacco Etch Virus protease (AcTEV) recognition site (ENLYFQG) was introduced by site directed mutagenesis (TSC1-TEV-myc).

To generate V5 epitope-tagged TSC2 C-terminal truncation constructs, XhoI sites were introduced into a V5-tagged full-length TSC2 expression construct [17] by site-directed mutagenesis. After excision of the 3' portions of the *TSC2* ORF with XhoI, the plasmids were re-circularised, leaving the epitope tag in-frame with the truncated ORF. To generate N-terminal truncations, BamHI sites were introduced and the 5' portion of the *TSC2* ORF excised, leaving a truncated ORF with a new translation initiation codon. Expression constructs were derived for 3 C-terminal truncations: A614V5 (TSC2 amino acids 1 – 614, F904V5 (amino acids 1 – 904) and A1153V5 (amino acids 1 – 1153), and 2 N-terminal truncations: M1028V5 (amino acids 1028 – 1784) and M1453V5 (amino acids 1453 – 1784). Other constructs used in this study have been described elsewhere [18]. All constructs were verified by sequence analysis of the entire ORF.

Antibodies were purchased from Cell Signaling Technology (Danvers, MA, U.S.A.) (mouse anti-myc, rabbit anti-myc, rabbit anti-TOR, rabbit anti-TSC2, rabbit anti-T1462 phosphorylated TSC2, rabbit anti-AKT), Roche Molecular Biochemicals (Basel, Switzerland) (mouse anti-GFP), Invitrogen (mouse anti-V5, mouse anti-Xpress), Santa Cruz Biotechnology (rabbit anti-14-3-3ζ) (Santa Cruz, CA, U.S.A.) or were described previously [5,19]. Secondary antibodies for the infra-red detection of blotted proteins were obtained from Li-Cor Biosciences (Lincoln, NE, U.S.A.). Blotted protein levels were estimated by near infra-red detection and quantification on an Odyssey^TM^ scanner (Li-Cor Biosciences).

### Subcellular fractionation

HEK 293T cells were washed and scraped into ice-cold phosphate buffered saline (PBS), pelleted by centrifugation (3000 g, 5 minutes, 4°C), resuspended in hypotonic buffer [12](10 mM HEPES pH 7.5, 10 mM KCl, 1.5 mM MgCl_2_, 0.1 mM EGTA, 20 mM NaF, plus protease inhibitors (Complete EDTA-free, Roche Molecular Biochemicals)) and drawn repeatedly through a fine-gauge hypodermic needle. Crude nuclei and undisrupted cells were removed by centrifugation (3000 g, 5 minutes, 4°C) and the supernatant was subjected to ultracentrifugation (100 000 g, 1 hour, 4°C). The supernatant, containing cytosolic proteins, was recovered and subjected to gel filtration. The pellet was resuspended in extraction buffer (50 mM HEPES pH 7.5, 10% v/v glycerol, 1 mM DTT, 100 mM NaCl, 10 mM NaF, 50 mM β-glycerophosphate, plus protease inhibitors (Complete, Roche Molecular Biochemicals)) to extract additional proteins and disrupt any weak salt-sensitive interactions. After ultracentrifugation (100 000 g, 1 hour, 4°C), the supernatant (100 mM NaCl extract) was subjected to gel filtration. The pellet was resuspended in extraction buffer containing 0.1% w/v Triton X100 and, after centrifugation at 10 000 g for 10 minutes at 4°C, the supernatant was subjected to gel filtration.

### SMART gel filtration

Gel filtration was performed on the SMART fast protein liquid chromatography apparatus (GE Healthcare) using a Superose 6 column. The column was pre-equilibrated for 30 minutes prior to the injection of 50 μl cell extract. Flowrate was set at 50 μl/min and the elution profile monitored at 280 nm. Elution fractions (50 μl) were collected and analysed by immunoblotting and compared to a series of molecular size markers: blue dextran (2 MDa), thyroglobulin (660 kDa), ferritin (400 kDa) and catalase (240 kDa). Gel filtration was carried out in 50 mM HEPES pH 7.5, 1 mM DTT, 100 mM NaCl, 10 mM NaF, 50 mM β-glycerophosphate, 1 mM EDTA. For analysis of the detergent extract, 0.1% w/v Triton X100 was included in the gel filtration buffer.

### Immunoprecipitation

HEK 293T cells in 6 cm dishes were transfected with expression constructs using polyethylenimine (Polysciences Inc., Warrington, PA, U.S.A.)[20]. Twenty-four hours after transfection the cells were washed with ice-cold PBS and lysed in 0.3 ml lysis buffer (50 mM HEPES pH 7.5, 10% v/v glycerol, 1 mM DTT, 100 mM NaCl, 10 mM NaF, 50 mM β-glycerophosphate, 0.1% Triton X100 plus protease inhibitors (Complete, Roche Molecular Biochemicals)) for 10 minutes on ice. The lysates were cleared by centrifugation (10 000 g for 10 minutes at 4°C).

For immunoprecipitation of myc or V5 tagged proteins, EZ Red anti-myc or anti-V5 affinity beads (Sigma-Aldrich) were pre-washed with lysis buffer and then incubated with the cleared lysates for 3 hours at 4°C with gentle rotation. For immunoprecipitations using antibodies against TSC2 or the Xpress tag, the antibodies were incubated with the lysates on ice for 90 minutes before transfer to pre-washed Protein A/G beads and incubation at 4°C for 3 hours with gentle rotation. Beads were washed at least 3 times with >20-fold excess of lysis buffer per wash, recovered between each wash by centrifugation (1000 g for 15 seconds at 4°C) and resuspended in sample buffer prior to immunoblot analysis.

### Affinity purification of the TSC1-TSC2 complex

HEK 293T cells in 10 cm dishes were cotransfected with the TSC2 and TSC1-TEV-myc expression constructs. Forty-eight hours after transfection the cells were rinsed with cold PBS and lysed in 0.4 ml of lysis buffer for 10 minutes on ice prior to centrifugation (10 000 g for 10 minutes at 4°C). The supernatant was transferred to 20 μl of a 50% suspension of EZ Red anti-myc affinity beads (Sigma-Aldrich), pre-equilibrated with lysis buffer, and agitated gently for 4 hours at 4°C. Beads were recovered by centrifugation (1000 g for 15 seconds at 4°C) and washed 3 times with 0.4 ml lysis buffer. The washed beads were resuspended in 40 μl of lysis buffer and incubated overnight at 4°C with 10U of AcTEV (Invitrogen). Beads were removed by centrifugation (1000 g for 15 seconds at 4°C) and the supernatant fraction analysed by gel filtration.

## Results and discussion

### Gel filtration of the TSC1-TSC2 complex

**Figure 1:**
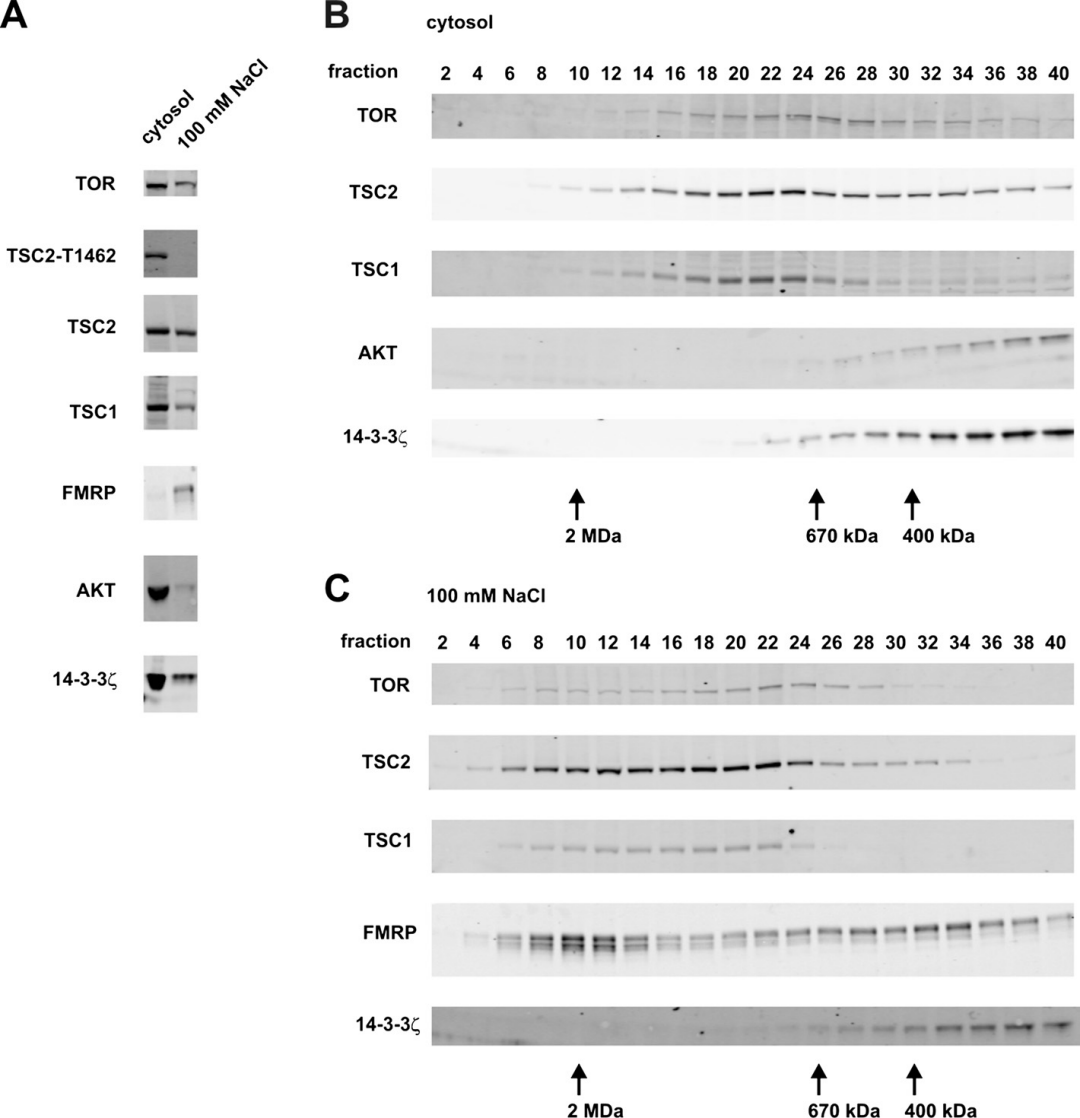
**Superose 6 gel filtration analysis of the TSC1-TSC2 complex.** (**A**) Subcellular fractionation of the TSC1-TSC2 complex. HEK 293T cells were disrupted as described in Materials and Methods, to prepare a hypotonic extract (cytosol) and a 100 mM NaCl extract. The distribution of TSC1, TSC2 and T1462-phosphorylated TSC2 (TSC2-T1462) in the 2 extracts was compared to the proteins TOR, FMRP, AKT and 14-3-3ζ by immunoblotting. (**B**) The cytosolic extract was applied to a Superose 6 gel filtration column and the elution fractions analysed by immunblotting for the presence of TOR, TSC2, TSC1, AKT and 14-3-3ζ. Peak elution fractions of the molecular weight standards are indicated. (**C**) The 100 mM NaCl extract was separated and analysed as in (B), except that the elution fractions were tested for FMRP instead of AKT.

Previously, we estimated the size of the TSC1-TSC2 complex by gel filtration of detergent lysates of HeLa cells [15]. TSC1 and TSC2 co-eluted at an estimated molecular weight > 450 kDa. More recently, we repeated these gel filtration experiments using detergent-free extracts of human embryonal kidney (HEK) 293T cells. Cells were first lysed in hypotonic buffer to prepare a cytosolic extract. Subsequently, the remaining insoluble material was extracted first with buffer containing 100 mM NaCl and finally in buffer containing 0.1% w/v Triton X100. Elution fractions of the extracts were collected and separated by SDS-PAGE and probed for the presence of TSC1 and TSC2 by immunoblotting, as shown in Figure 1. For comparison, and to confirm that the lysis and gel filtration procedure gave the expected results for other proteins, we screened the elution fractions for TOR, AKT, FMRP and 14-3-3ζ. TSC1 and TSC2 were present in all extracts, similar to TOR and 14-3-3ζ. In contrast, AKT was predominantly in the cytosolic extract, as expected [21], while most FMRP was detected in the 100 mM NaCl extract.

TSC1 (130 kDa) and TSC2 (200 kDa) co-eluted across a broad range of fractions. For both proteins the peak fractions were 20–24 (estimated molecular weight 900–1200 kDa) for the cytosolic extract, and 18–22 (1100–1400 kDa) for the 100 mM NaCl extract (Figure 1 and Additional file 2B and E). The elution profiles for TOR were slightly different to those for TSC1 and TSC2. TOR eluted slightly later, with peak fractions 26–28 (600–800 kDa) for the cytosolic extract and 24–28 (600–900 kDa) for the 100 mM NaCl extract (Figure 1 and Additional file 2A and D), corresponding quite well with previous estimates of the size of TOR complexes (~700 kDa) [22-24]. AKT (60 kDa) and 14-3-3ζ (30 kDa) eluted later, with fractions 38 and 40 (< 200 kDa) showing the strongest signals (Figure 1 and Additional file 2C and F). The elution profile for FMRP had 2 distinct peaks, corresponding to fractions 10–12 (1800–2000 kDa) and 32–34 (200–300 kDa). Most likely the FMRP detected in fractions 10–12 corresponds to FMRP associated with ribosomes, while the peak corresponding to fractions 32–34 represents FMRP homo-multimers [25].

The combined molecular weight of the TSC1-TSC2 dimer is 330 kDa. Therefore, the elution profiles suggested that TSC1 and TSC2 exist as subunits of larger protein complexes, consistent with previous findings [15,16]. TSC2 was detected in a broader range of fractions than TSC1, particularly from the cytosolic extract (Figure 1B and Additional file 2B), suggesting that a proportion of the TSC2 in the cytosol is not associated with TSC1.

The TSC1-TSC2 complex is phosphorylated at multiple sites, including T1462, by AKT, resulting in inhibition of TSC1-TSC2 complex function. It has been proposed that AKT-dependent phosphorylation of TSC2 and subsequent binding of 14-3-3 proteins results in sequestration of TSC1-TSC2 complexes in the cytosol [12], and therefore in a distinct compartment from the TSC1-TSC2 substrate RHEB which is predominantly active on endomembranes [26]. Consistent with this idea, we only detected T1462-phosphorylated TSC2 in fractions from the cytosolic extract (Figure 1). Furthermore, although we did not demonstrate a direct interaction between TSC2 and 14-3-3ζ, co-elution of TSC2 and 14-3-3ζ in fractions 24–40 of both extracts (Figure 1 and Additional file 2) is consistent with TSC2-14-3-3ζ binding [27,28]. The elution profiles of TOR, AKT, FMRP and 14-3-3ζ were consistent with previous findings and therefore it is unlikely that the apparent large size estimates for the TSC1-TSC2 complex in the cytosolic and 100 mM NaCl extracts were artefacts of the lysis and fractionation procedure.

TOR, TSC1 and TSC2 co-eluted in the early fractions of the Triton X100 extract (Additional file 2G and H; peak fraction 10, estimated molecular weight ~2 MDa). One possibility is that TOR and TSC1-TSC2 complexes associate with detergent-containing micelles. Alternatively, these proteins may form very large (≥ 2 MDa), insoluble, membrane-associated complexes. We did not account for the formation of these structures in our original investigation [15], and it is possible that this may partly explain the differences between our previous and current estimates for the molecular weight of the TSC1-TSC2 complex. Nonetheless, in both cases, the estimated molecular weight of the complex is greater than could be accounted for by a 1:1 heterodimer, consistent with the presence of multiple TSC1 and/or TSC2 subunits in the TSC1-TSC2 complex. Interestingly, the elution profiles of TSC1 and TSC2 are similar to those described for the RGC1-RGC2/AS250, RalGAP1 and RalGAP2 GAP complexes [29-31]. In each case, the active GAP complex consists of two distinct subunits, of which the catalytic subunit has significant homology with the TSC2 GAP-domain [30-32]. It is possible that such multimeric GAP complexes would be more responsive allosteric regulation [13,14], and therefore more sensitive to changes in upstream signalling events.

Next, we determined the elution profile of affinity purified TSC1-TSC2 complexes. Coomassie staining of the purified complex revealed equal amounts of TSC1 and TSC2 and no other major protein components (Additional file 3), consistent with previous results [32]. Affinity purified TSC1 and TSC2 co-eluted (peak fraction 20; 1200 kDa), indicating that TSC1 and TSC2 form high molecular weight complexes in the absence of other protein components. In agreement with a previous report [16], we concluded that these complexes contain multiple TSC1 and TSC2 subunits, possibly as many as a 4:4 heterooctamer (predicted molecular weight 1320 kDa). The presence of several co-occuring TSC1 and TSC2 peaks in the elution profile suggests that TSC1 and TSC2 can assemble into multiple distinct complexes, containing different numbers of TSC1 and TSC2 subunits.

### Coimmunoprecipitation of TSC1-TSC2 complexes

**Figure 2:**
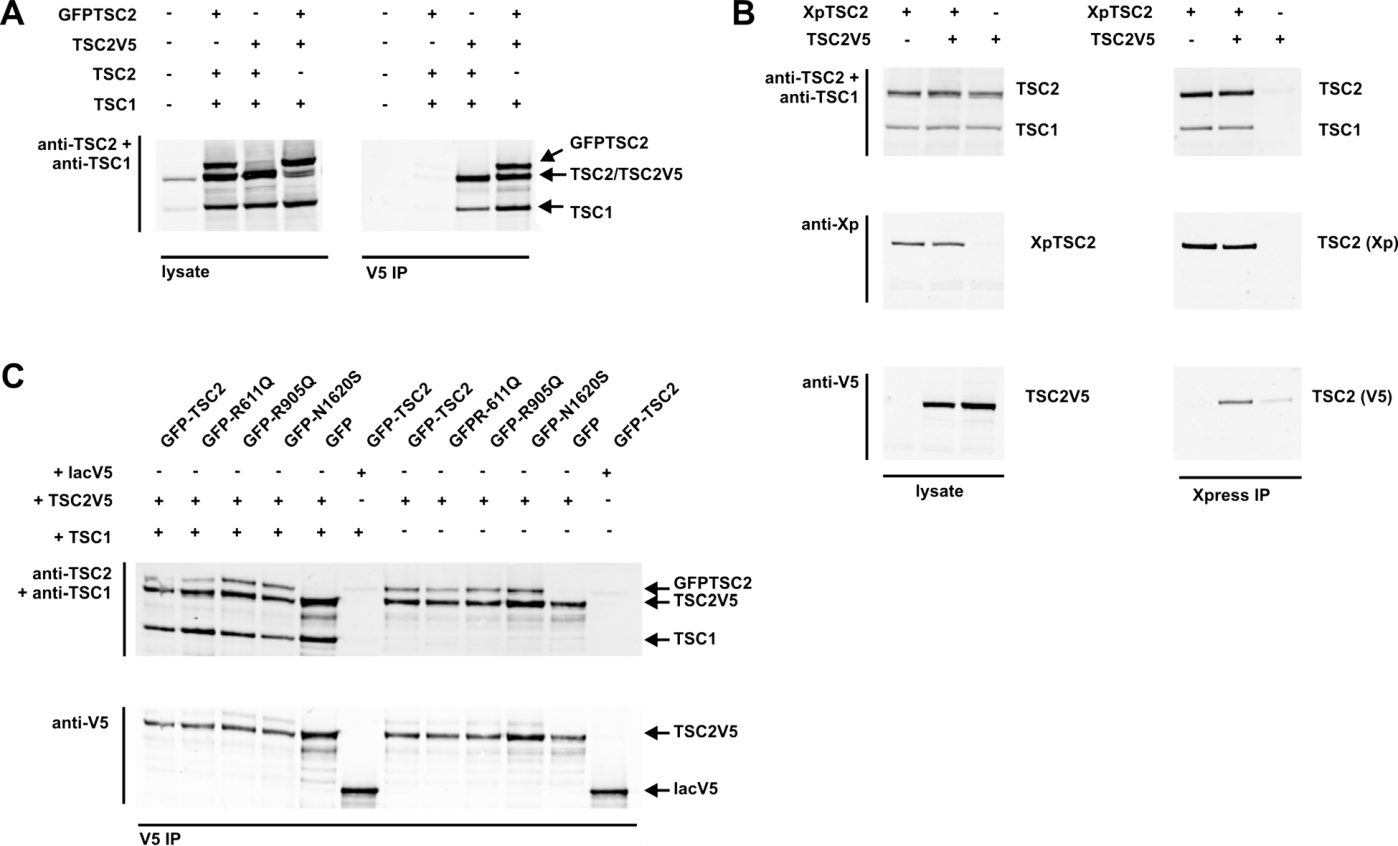
**Coimmunoprecipitation of distinct epitope-tagged TSC2 isoforms.** Untagged TSC2, GFPTSC2, XpTSC2 and/or TSC2V5 were co-expressed in HEK293T cells with or without co-expressed TSC1. Distinct tagged isoforms were immunoprecipitated and the presence of other coimmunoprecipitated tagged isoforms was investigated by immunoblotting. (**A**) Coimmunoprecipitation of TSC1 and GFPTSC2 with TSC2V5. TSC2V5 was co-expressed with GFPTSC2 or untagged TSC2. GFPTSC2 was detectable as a 220 kDa band in immunoblots of the cell lysates, distinct from the 200 kDa TSC2 and TSC2V5 bands. TSC2V5 was immunoprecipitated from the lysates with V5 affinity beads. Immunoblotting of the washed beads showed that GFPTSC2 was coimmunoprecipitated with TSC2V5. No GFPTSC2 was immunoprecipitated in the absence of TSC2V5. (**B**) Coimmunoprecipitation of TSC1 and TSC2V5 with XpTSC2. XpTSC2 was immunoprecipitated from cell lysates with an antibody specific for the Xpress tag. The Xpress- and V5-tagged TSC2 isoforms could be distinguished by immunoblotting using the appropriate specific antibodies. TSC2V5 coimmunoprecipitated with XpTSC2. (**C**) Coimmunoprecipitation of GFPTSC2 variants with TSC2V5. TSC2V5 was co-expressed with GFPTSC2 or GFPTSC2 containing the R611Q (GFPR611Q), R905Q (GFPR905Q) or N1620S (GFPN1620S) pathogenic substitutions, with or without co-expressed TSC1. As a control, GFPTSC2 was co-expressed with V5-tagged B-lactamase (lacV5). All the GFP-tagged TSC2 variants were coimmunoprecipitated with TSC2V5. In contrast, GFPTSC2 was not detected in the lacV5 immunoprecipitates.

To confirm the existence of TSC1-TSC2 complexes containing multiple TSC1 and TSC2 subunits, we coexpressed distinct epitope tagged TSC1 and TSC2 isoforms and determined the composition of TSC1-TSC2 complexes containing these isoforms by immunoprecipitation. Immunoprecipitation of TSC2V5 with anti-V5 antibodies resulted in coimmunoprecipitation of GFPTSC2, as well as TSC1 (Figure 2A). Similarly, TSC2V5 was coimmunoprecipitated with N-terminal Xpress epitope tagged TSC2 (XpTSC2) and anti-Xpress antibodies (Figure 2B).

Specific missense mutations in the N-terminal region of TSC2, such as the R611Q substitution [18], disrupt the TSC1-TSC2 interaction. Other pathogenic TSC2 amino acid substitutions, such as N1620S, most likely inactivate the TSC1-TSC2 complex by altering the conformation of the active site in the GAP domain without affecting TSC1-TSC2 complex formation [33]. Finally, other pathogenic mutations, such as R905Q, clearly affect TSC2 function [34], although it is not yet clear how. We investigated whether GFPTSC2-TSC2V5 coimmunoprecipitation was affected by the co-expression of TSC1 or by the introduction of the pathogenic TSC2 R611Q, R905Q or N1620S substitutions. As shown in Figure 2C, neither the absence of TSC1, nor the introduction of the R611Q, R905Q or N1620S mutations affected GFPTSC2-TSC2V5 coimmunoprecipitation. Therefore, we concluded that TSC1 is not required for the TSC2-TSC2 interaction and that this interaction is direct and not affected by the R611Q, R905Q or N1620S mutations.

**Figure 3:**
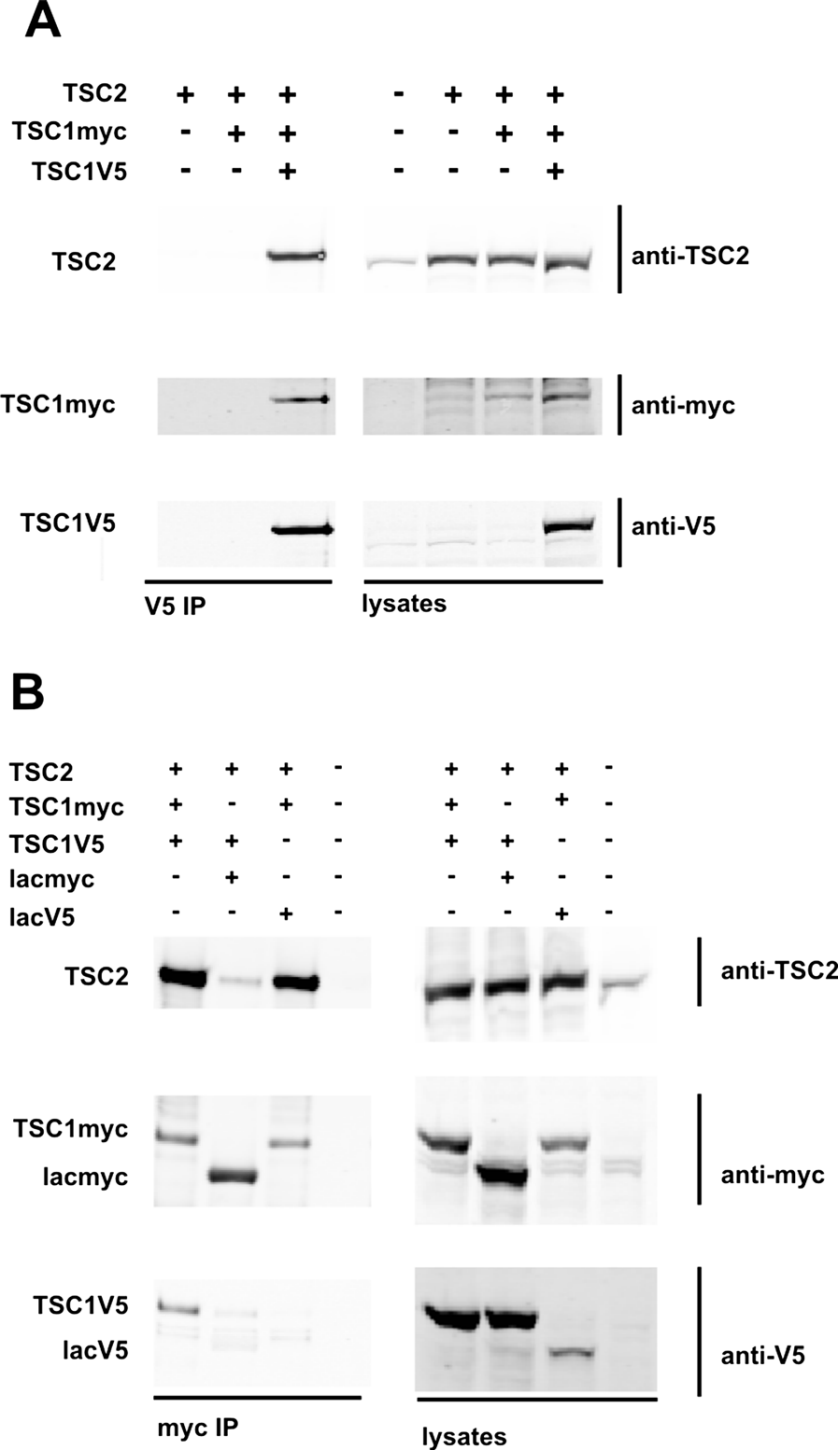
**Coimmunoprecipitation of distinct epitope-tagged TSC1 isoforms.** TSC1myc and TSC1V5 were co-expressed with TSC2 in HEK293T cells. The distinct tagged TSC1 isoforms were immunoprecipitated and the presence of other coimmunoprecipitated tagged isoforms was investigated by immunoblotting. (**A**) Coimmunoprecipitation of TSC2 and TSC1myc with TSC1V5. TSC1V5 was co-expressed with TSC2 and TSC1myc. TSC1V5 was immunoprecipitated with anti-V5 affinity beads, washed and the immunoprecipitates analysed by immunoblotting. TSC1myc and TSC2 were detected in the TSC1V5 immunoprecipitate. (**B**) Coimmunoprecipitation of TSC2 and TSC1V5 with TSC1myc. TSC1myc was co-expressed with TSC2 and TSC1V5 or V5-tagged β-lactamase (lacV5). TSC1myc was immunoprecipitated with anti-myc affinity beads, washed and the immunoprecipitates analysed by immunoblotting. TSC1V5 and TSC2, but not lacV5, were detected in the TSC1myc immunoprecipitate. As an extra control, TSC2 and TSC1V5 were co-expressed with myc-tagged β-lactamase (lacmyc), and immunoprecipitated with anti-myc affinity beads. TSC1V5 was not detected in the lacmyc immunoprecipitate.

Over-expression of TSC1 in the absence of co-expressed TSC2 results in the formation of large, insoluble aggregates of TSC1. Co-expression of TSC2 prevents TSC1 aggregation due to the formation of soluble TSC1-TSC2 complexes [5,15]. To determine whether these soluble TSC1-TSC2 complexes contain multiple TSC1 molecules, we coexpressed TSC1 tagged with a C-terminal V5 epitope (TSC1V5) and TSC1 tagged with a C-terminal myc epitope (TSC1myc) with TSC2. Immunoprecipitation of TSC1V5 resulted in coimmunoprecipitation of TSC1myc and TSC2, and TSC1V5 and TSC2 were coimmunoprecipitated with TSC1myc (Figure 3).

### The N- and C- terminal regions of TSC2 are involved in the TSC2-TSC2 interaction

**Figure 4:**
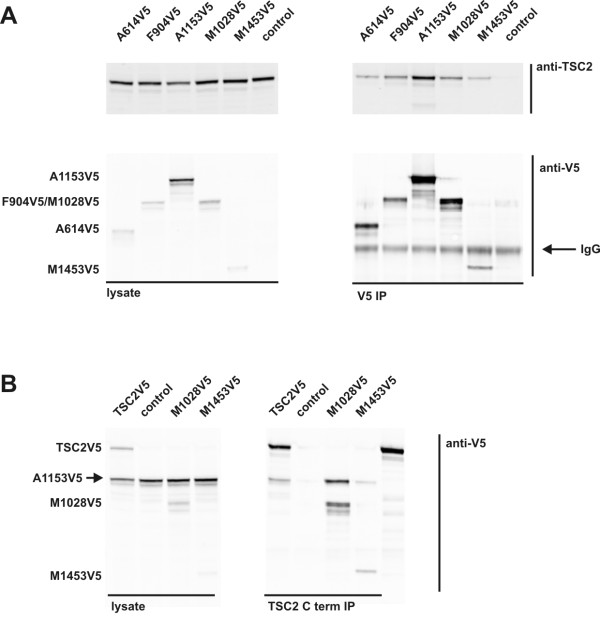
**Coimmunoprecipitation analysis of TSC2-TSC2 interactions using truncated TSC2 proteins.** A series of V5-tagged TSC2 truncation proteins were coexpressed with TSC2, or in combination. The truncated proteins were immunoprecipitated and coimmunoprecipitated proteins detected by immunoblotting. (**A**) Coimmunoprecipitation of untagged, full-length TSC2 with TSC2 truncation proteins. The V5-tagged truncated TSC2 proteins (A614V5, F904V5, A1153V5, M1028V5 and M1453V5; see Materials and Methods and Suppl. Figure 1. for details) were immunoprecipitated with anti-V5 affinity beads and the washed immunoprecipitates were analysed by immunoblotting. TSC2 was coimmunoprecipitated with all 5 TSC2 truncation proteins, but was not visible in the control transfection (control; no V5-tagged protein expressed). (**B**) Coimmunoprecipitation of the A1153V5 TSC2 truncation protein with untagged TSC2 and the N-terminal TSC2 truncation proteins, M1028V5 and M1453V5. Full-length TSC2 and the M1028V5 and M1453V5 truncation proteins were immunoprecipitated with a polyclonal antibody recognising epitopes encoded by the last exon of TSC2 [5] and the washed immunoprecipitates were analysed by immunoblotting. In each case, the A1153V5 truncation protein was coimmunoprecipitated. No A1153V5 was visible in the immunoprecipitate of control cells, expressing A1153V5 only (control).

To investigate the TSC1-TSC2 and TSC2-TSC2 interactions in more detail, we performed coimmunoprecipitation experiments using a series of truncated TSC2 proteins. Consistent with previous findings [15], we found that the N-terminal region of TSC2 was required for binding TSC1 (Additional file 4). TSC1myc was coimmunoprecipitated with truncation proteins consisting of TSC2 amino acids 1–614 (TSC2 A614V5), 1–904 (TSC2 F904V5) or 1–1153 (TSC2 A1153V5) but not with truncation proteins consisting of TSC2 amino acids 1028–1784 (TSC2 M1028V5) or 1453–1784 (TSC2 M1453V5). In contrast, TSC2 was coimmunoprecipitated by all the TSC2 truncation proteins tested (Figure 4). Furthermore, the A1153V5 truncation coimmunoprecipitated with the M1028V5 and M1453V5 truncations, indicating that the N-terminal and C-terminal regions of TSC2 are both involved in TSC2-TSC2 binding.

## Conclusions

We performed gel filtration and coimmunoprecipitation experiments to investigate the quaternary structure of the TSC1-TSC2 complex. Although we could not confirm our previous estimate of the size of the TSC1-TSC2 complex, our new data support the proposal that the TSC1-TSC2 complex can contain multiple TSC1 and TSC2 subunits [15,16]. Classical enzyme kinetic models indicate that multimeric complexes can be very sensitive to the effects of allosteric interactions, and therefore better regulators [13]. A multimeric TSC1-TSC2 complex would potentially be more responsive to changes in upstream signalling events than the single heterodimer.

Our data indicate that the N- and C-terminal regions of TSC2 are involved in the TSC2-TSC2 interaction, and that TSC1 is not required for TSC2-TSC2 binding. Further studies will be required to elucidate the exact nature of the inter-molecular interactions that stabilise the TSC1-TSC2 complex, and to determine whether oligomerisation of the TSC1-TSC2 heterodimer is important for the regulation of TORC1 signalling in normal and disease states. In particular it will be interesting to determine whether specific, pathogenic TSC1 and TSC2 amino acid substitutions affect TSC1-TSC2 oligomerisation.

## Abbreviations

AcTEV: Tobacco etch virus protease; AKT: Protein kinase B; FMRP: Fragile X mental retardation protein; GAP: GTPase activating protein; GFP: Green fluorescent protein; HEK: Human embryonal kidney; LAM: Lymphangioleiomyomatosis; RHEB: Ras homolog expressed in brain; TOR: Target of rapamycin; TORC1: TOR complex 1; TSC: Tuberous sclerosis complex.

## Competing interests

The authors declare that they have no competing interests.

## Authors’ contributions

MHW, LvU, AH and MN performed the experimental work. All authors assisted with data analysis and interpretation, and read and approved the final manuscript.

## Supplementary Material

Additional file 1 **TSC1 and TSC2 truncation proteins.** Schematic diagram illustrating the epitope tagged and truncated TSC1 and TSC2 isoforms used as part of this study. The different expressed proteins are represented by bars scaled according to the number of encoded amino acids, compared to the untagged isoforms (top). GFP, Xpress (Xp), myc and V5 epitope tags are represented by solid (filled) regions at either the amino- (N) or carboxy- (C) terminal of the full-length and truncated proteins. The most C-terminal amino acids are indicated above the bars. (A) Epitope-tagged and truncated TSC2 isoforms. The position of the TSC2 GAP domain is indicated. The positions of the amino acid substitutions in the GFPTSC2 isoform, and the initiation methionines for the 2 N-terminal TSC2 truncation proteins are shown above the bars. (B) Epitope-tagged TSC1 isoforms. The positions of the putative transmembrane domain and coiled coil region are indicated*.* The location of the AcTEV endonuclease cleavage site is shown (AcTEV). Click here for file

Additional file 2 **Overview of the Superose 6 gel filtration experiments.** The integrated intensities of the protein bands in the different elution fractions were determined in 3 separate immunoblot experiments. The total signal per protein was determined and the relative signal per fraction was calculated. Elution profiles for TOR (A, D and G), TSC1 and TSC2 (B, E and H), AKT (C), FMRP (F) and 14-3-3ζ (C and F) from the cytosolic (A - C), 100 mM NaCl (D - F) and 0.1% Triton X100 (G - H) extracts are shown. Peak elution fractions of the molecular weight standards are indicated. Error bars show the standard error of the mean. Click here for file

Additional file 3 **Superose 6 gel filtration of affinity purified TSC1-TSC2 complexes.** (A) TSC1-TSC2 complexes released from anti-myc affinity beads by AcTEV endonuclease digestion (see Materials and Methods for details) were resolved by SDS-PAGE and detected by Coomassie staining. Approximately equal quantities of both proteins were visible on the gels, and no other major protein components (>50 kDa in size) were detected. (B) The purified TSC1-TSC2 complexes were applied to the Superose 6 column and the elution fractions analysed by immunblotting. Peak elution fractions of the molecular weight standards are indicated. (C and D) Elution profiles for TSC2 (C) and TSC1 (D). The integrated intensity of the protein bands were determined per fraction. The peak elution fractions of the molecular weight standards are indicated. Click here for file

Additional file 4 **Coimmunoprecipitation of TSC1with the N-terminal of TSC2.** TSC1 was co-expressed with TSC2V5, the TSC2 truncation proteins (A614V5, F904V5, A1153V5, M1028V5 and M1453V5), V5-tagged β-lactamase (lacV5), or empty vector (control). The V5-tagged proteins were immunoprecipitated with anti-V5 affinity beads and the washed immunoprecipitates were analysed by immunoblotting. TSC1 was coimmunoprecipitated with TSC2V5 and with the C-terminal truncation proteins A614V5, F904V5 and A1153V5 (encoding amino acids 1–614, 1–904 and 1–1153 respectively), but not with the N-terminal truncation proteins M1028V5 and M1453V5 (encoding amino acids 1028–1784 and 1453–1784, respectively), or lacV5. Click here for file
