# Supplementary figures and images for: The TSC1-TSC2 complex consists of multiple TSC1 and TSC2 subunits
Source: BMC Biochem. 2012 Sep 24;13:18. doi: 10.1186/1471-2091-13-18 (PMC3517901; doi:10.1186/1471-2091-13-18)

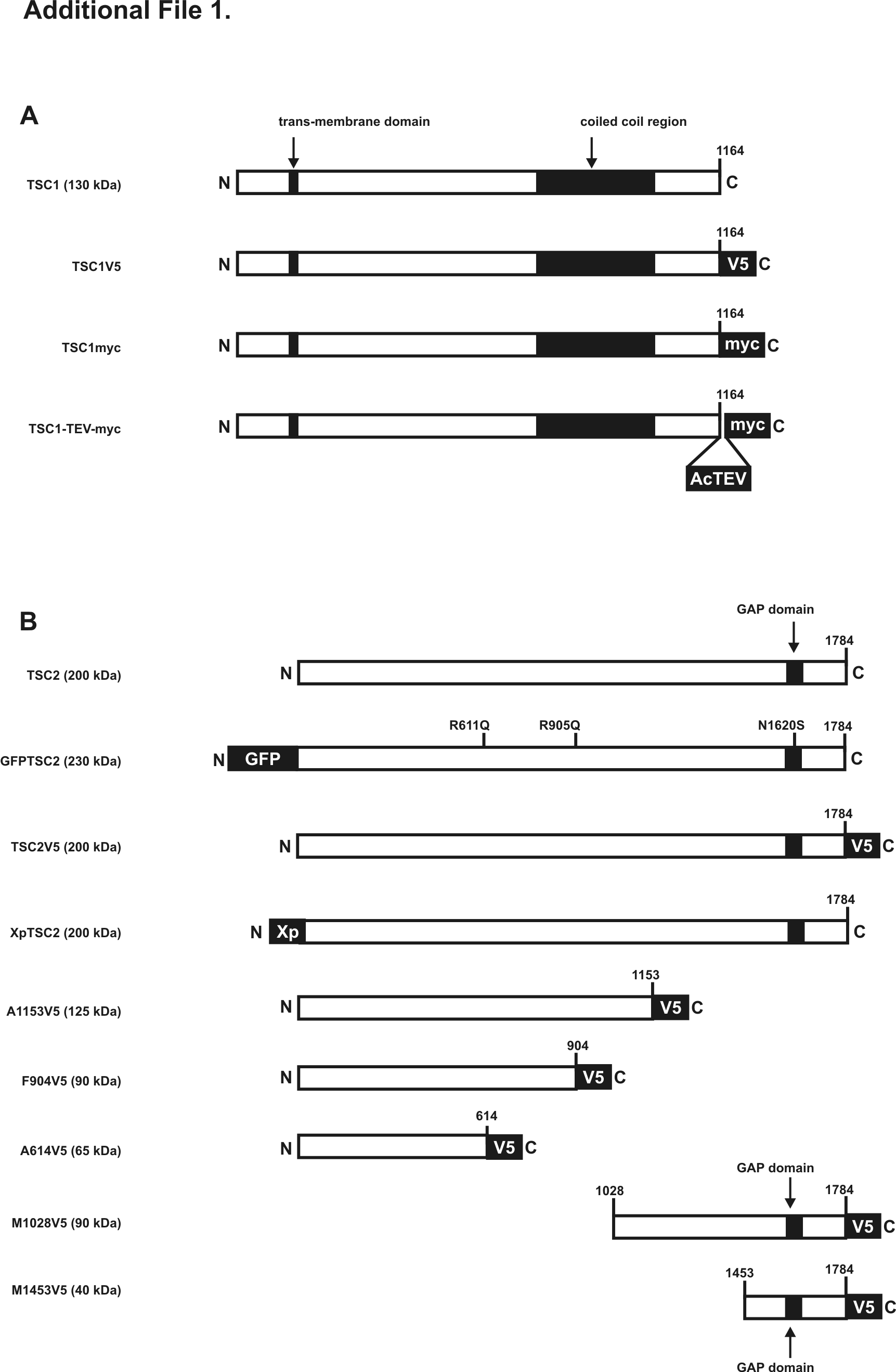

Supplement: Additional file 1 — TSC1 and TSC2 truncation proteins. Schematic diagram illustrating the epitope tagged and truncated TSC1 and TSC2 isoforms used as part of this study. The different expressed proteins are represented by bars scaled according to the number of encoded amino acids, compared to the untagged isoforms (top). GFP, Xpress (Xp), myc and V5 epitope tags are represented by solid (filled) regions at either the amino- (N) or carboxy- (C) terminal of the full-length and truncated proteins. The most C-terminal amino acids are indicated above the bars. (A) Epitope-tagged and truncated TSC2 isoforms. The position of the TSC2 GAP domain is indicated. The positions of the amino acid substitutions in the GFPTSC2 isoform, and the initiation methionines for the 2 N-terminal TSC2 truncation proteins are shown above the bars. (B) Epitope-tagged TSC1 isoforms. The positions of the putative transmembrane domain and coiled coil region are indicated. The location of the AcTEV endonuclease cleavage site is shown (AcTEV). [file 1471-2091-13-18-S1.tiff]

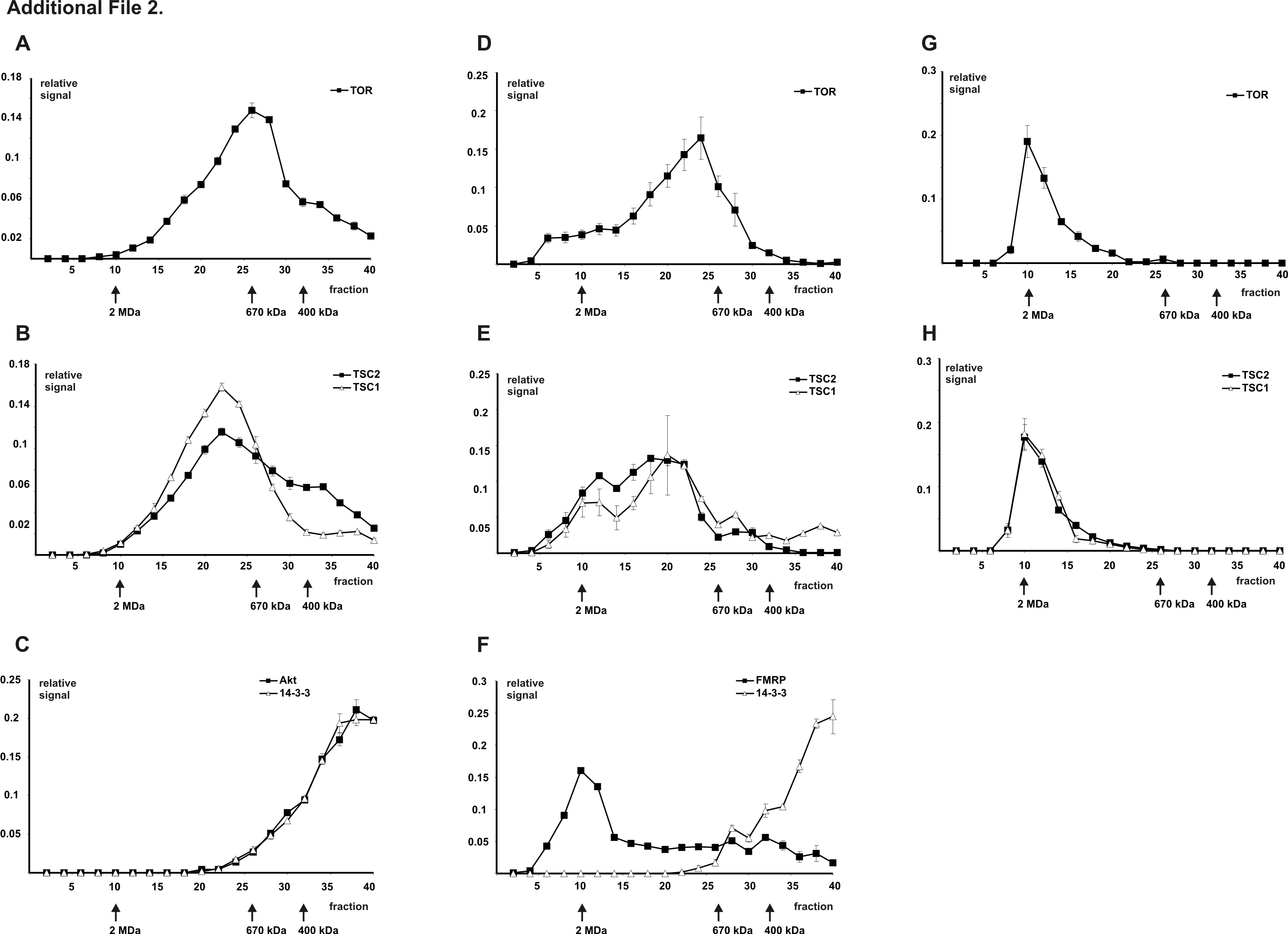

Supplement: Additional file 2 — Overview of the Superose 6 gel filtration experiments. The integrated intensities of the protein bands in the different elution fractions were determined in 3 separate immunoblot experiments. The total signal per protein was determined and the relative signal per fraction was calculated. Elution profiles for TOR (A, D and G), TSC1 and TSC2 (B, E and H), AKT (C), FMRP (F) and 14-3-3ζ (C and F) from the cytosolic (A - C), 100 mM NaCl (D - F) and 0.1% Triton X100 (G - H) extracts are shown. Peak elution fractions of the molecular weight standards are indicated. Error bars show the standard error of the mean. [file 1471-2091-13-18-S2.tiff]

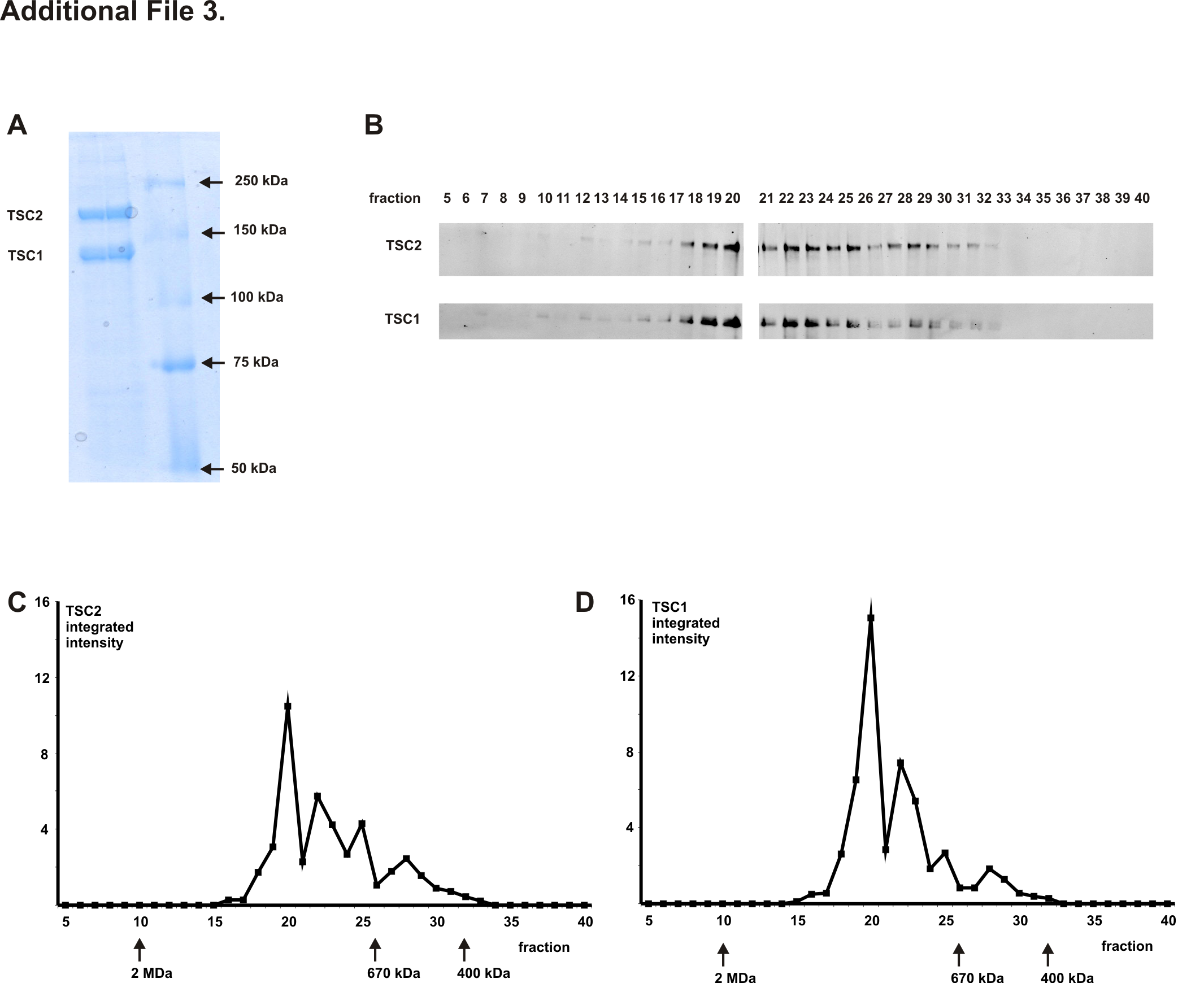

Supplement: Additional file 3 — Superose 6 gel filtration of affinity purified TSC1-TSC2 complexes. (A) TSC1-TSC2 complexes released from anti-myc affinity beads by AcTEV endonuclease digestion (see Materials and Methods for details) were resolved by SDS-PAGE and detected by Coomassie staining. Approximately equal quantities of both proteins were visible on the gels, and no other major protein components (>50 kDa in size) were detected. (B) The purified TSC1-TSC2 complexes were applied to the Superose 6 column and the elution fractions analysed by immunblotting. Peak elution fractions of the molecular weight standards are indicated. (C and D) Elution profiles for TSC2 (C) and TSC1 (D). The integrated intensity of the protein bands were determined per fraction. The peak elution fractions of the molecular weight standards are indicated. [file 1471-2091-13-18-S3.tiff]

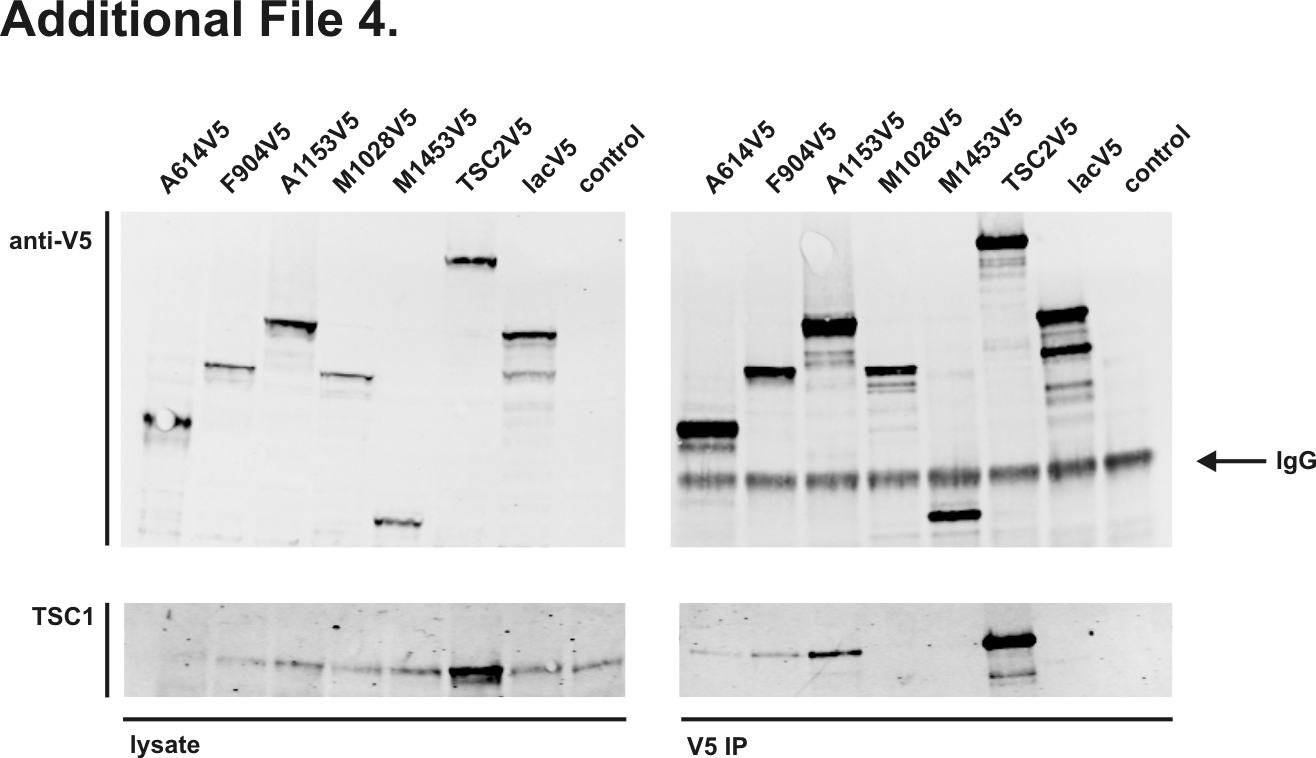

Supplement: Additional file 4 — Coimmunoprecipitation of TSC1with the N-terminal of TSC2. TSC1 was co-expressed with TSC2V5, the TSC2 truncation proteins (A614V5, F904V5, A1153V5, M1028V5 and M1453V5), V5-tagged β-lactamase (lacV5), or empty vector (control). The V5-tagged proteins were immunoprecipitated with anti-V5 affinity beads and the washed immunoprecipitates were analysed by immunoblotting. TSC1 was coimmunoprecipitated with TSC2V5 and with the C-terminal truncation proteins A614V5, F904V5 and A1153V5 (encoding amino acids 1–614, 1–904 and 1–1153 respectively), but not with the N-terminal truncation proteins M1028V5 and M1453V5 (encoding amino acids 1028–1784 and 1453–1784, respectively), or lacV5. [file 1471-2091-13-18-S4.tiff]
